# Point-of-care Ultrasound Diagnosis of Acute Sialolithiasis with Sialadenitis

**DOI:** 10.5811/cpcem.2017.7.34907

**Published:** 2017-10-18

**Authors:** Felix Huang, Rebecca Caton, Joseph Colla

**Affiliations:** University of Illinois at Chicago, Department of Emergency Medicine, Chicago, Illinois

## CASE PRESENTATION

**Image 1:**
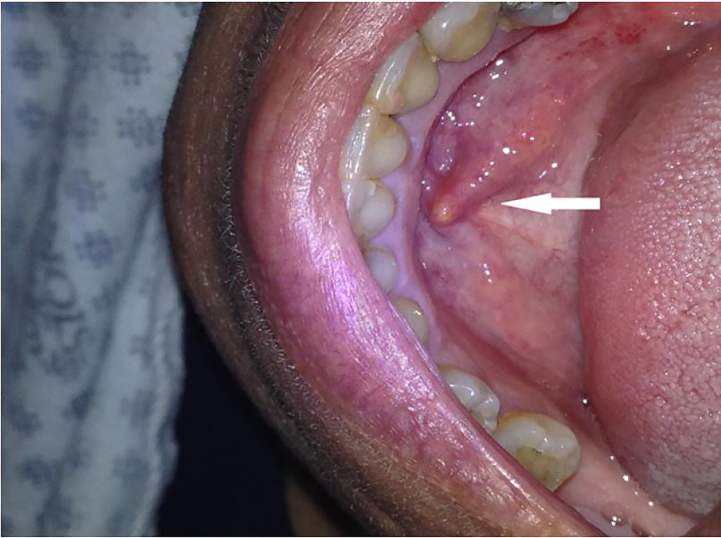
Right-sided submandibular swelling of Wharton’s duct (arrow).

**Image 2:**
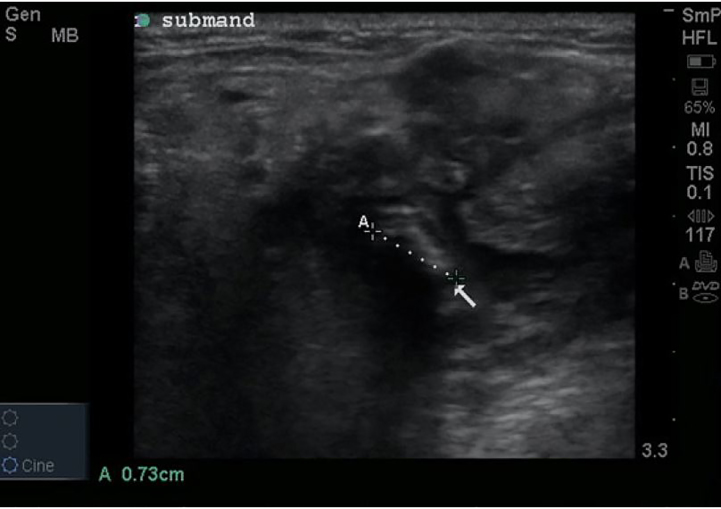
Ultrasound image of a 7.3 mm sialolith obstructing the salivary duct (arrow). Computed tomography confirmed an eight mm stone within the right Wharton’s duct (Image 3).

**Image 3:**
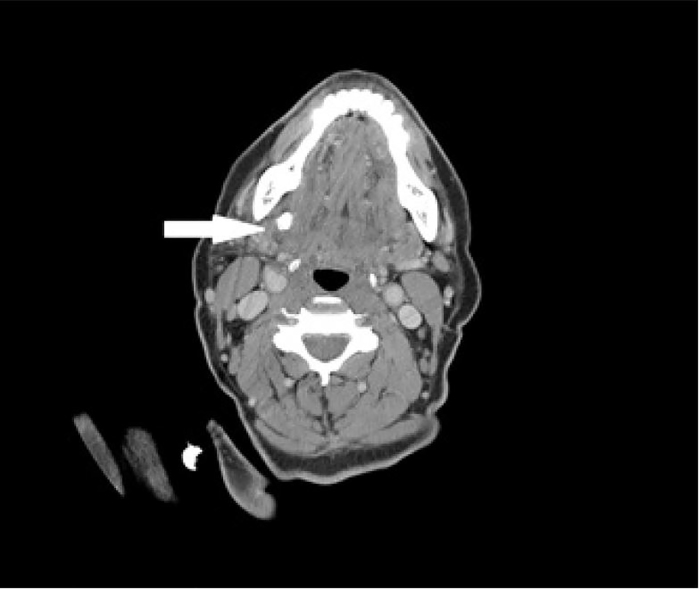
Computed tomography image of an eight millimeter stone within the right Wharton’s duct (arrow).

A 63-year-old female with a past medical history of gastroesophageal reflux disease, diabetes, and arthritis presented with right-sided jaw swelling for one day, radiating to the right ear, associated with some odynophagia. Vital signs were a tympanic temperature of 37.0 degrees Celsius, pulse rate of 91 beats per minute, respiratory rate of 20 breaths per minute, and a blood pressure 131/84 mmHg, saturating 96% on room air. Exam was significant for right submandibular swelling (Image 1) and mild tenderness and edema to the right posterior neck along the sternocleidomastoid. The physician performed a point-of-care ultrasound (POCUS) (Image 2) and subsequently ordered computed tomography (CT) (Image 3) to confirm the diagnosis.

## DISCUSSION

### Diagnosis

Acute sialolithiasis with sialadenitis. POCUS with a linear, high-frequency probe revealed an enlarged, hyperemic right submandibular gland with evidence of a 7.3 mm sialolith obstructing the salivary duct (Image 2). The ultrasound can be performed with the patient supine, the neck extended and head laterally rotated away from the side being examined. Place the high-frequency, linear probe in the submandibular region underneath the body of the mandible and scan along the mylohyoid muscle, anterior to the digastric muscle. The submandibular gland is best visualized in a slightly oblique plane and appears as a well-capsulated structure with a uniform parenchymal echo pattern. The duct will be a hypoechoic linear structure with a thin echogenic wall that lies medial to the sublingual gland.^1^ Sialoliths will appear as an echogenic structure with posterior acoustic shadowing.

CT has traditionally been the diagnostic modality to identify undifferentiated jaw swelling, with high sensitivity at identifying both sialolithiasis and sialadenitis as well as other etiologies in the differential diagnosis such as salivary gland tumors, other malignancies and abscesses.^1^ However, ultrasound is an attractive first-line diagnostic modality with a sensitivity of greater than 90% for stones greater than two millimeters and can be done at the bedside without ionizing radiation.^2^ Classic findings of sialolithiasis include hyperechoic bodies with acoustic shadowing representing stones.

The patient followed up with otolaryngology as an outpatient three days later with subsequent removal of the sialolith 14 days later in the operating room. There were no complications from the procedure.

Our case highlights the utility of POCUS to facilitate the diagnosis of submandibular sialolithiasis and sialadenitis by an emergency physician in the ED. We believe that POCUS can provide valuable information quickly without radiation exposure to the patient and that CT or other imaging techniques can be reserved for those patients with inconclusive ultrasounds or where complications such as tumors or abscesses may be suspected.

**CPC-EM Capsule**

What do we already know about this clinical entity?

Sialolithiasis refers to the formation of calculi within the salivary gland, and sialadenitis refers to the inflammation that occurs when the gland is obstructed.

What is the major impact of the image(s)?

Diagnosis of sialolithiasis can be confirmed by point-of-care ultrasound performed by emergency physicians.

How might this improve emergency medicine practice?

Bedside ultrasound is a rapid, non-ionizing, first-line method to confirm diagnosis when suspicion of other complications such as abscess is low.
